# Investigation of the Fermentation Process of *Moringa oleifera* Leaves and Its Effects on the Growth Performance, Antioxidant Capacity, and Intestinal Microbiome of *Procambarus clarkii*

**DOI:** 10.3390/antiox13111355

**Published:** 2024-11-05

**Authors:** Zhengzhong Li, Weizhu Luo, Qunlan Zhou, Cunxin Sun, Xiaochuan Zheng, Bo Liu, Kaunda Mpange, Aimin Zhu, Aimin Wang

**Affiliations:** 1 Key Laboratory of Freshwater Fisheries and Germplasm Resources Utilization, Ministry of Agriculture and Rural Affairs, Freshwater Fisheries Research Center, Chinese Academy of Fishery Sciences, Wuxi 214081, China; ymlizz@163.com (Z.L.); lwz13331041@163.com (W.L.); zhouql@ffrc.cn (Q.Z.); suncx@ffrc.cn (C.S.); zhengxiaochuan@ffrc.cn (X.Z.); 2 Wuxi Fisheries College, Nanjing Agricultural University, Wuxi 214081, China; 3 Department of Fisheries, Ministry of Fisheries and Livestock, Lusaka 10101, Zambia; mpangekaunda90@gmail.com; 4 Yancheng Academy of Fishery Sciences, Yancheng 224051, China; zam--3@163.com; 5 College of Marine and Bioengineering, Yancheng Institute of Technology, Yancheng 224007, China; blueseawam@ycit.cn

**Keywords:** fermentation process, growth performance, antioxidant capacity, intestinal microbiome, *M. oleifera*, crayfish

## Abstract

*Moringa oleifera* is renowned for its high antioxidant activity. However, few studies have been conducted on its effects on aquatic animals. The aim of this experiment was to investigate the optimal fermentation process of *M. oleifera* leaves and to evaluate the effects of fermented *M. oleifera* leaves on crayfish (9.11 ± 0.3 g) in terms of growth performance, antioxidant capacity, and gut microbiological parameters. By optimizing the fermenting material/water ratio, fermentation time, temperature, and strain, the optimal fermentation conditions of a 10% water ratio + 48 h + 30 °C + inoculation with 2% *B. amyloliquefaciens* (10^7^ CFU mL^−1^) were obtained. These conditions resulted in notable increases in the contents of the total protein, total phenols, flavonoids, and amino acids (*p* < 0.05) while also leading to a notable decrease in the content of tannins in contrast to those of unfermented *M. oleifera* leaves (*p* < 0.05). The fermented *M. oleifera* (FMO) leaves were incorporated at five concentrations, including 0% (control (CT)), 0.25% (0.25FMO), 0.5% (0.5FMO), 1% (1FMO), and 2% (2FMO). The results showed that the 1FMO group performed better in terms of the final body weight (FBW), weight gain rate (WGR), and specific weight gain rate (SGR) compared with the CT group (*p* < 0.05). In addition, amylase and lipase activities were significantly higher in the 1FMO and 2FMO groups compared with the other groups (*p* < 0.05). The fermented *M. oleifera* leaves significantly increased the catalase (CAT) activity in the crayfish (*p* < 0.05). The superoxide dismutase (SOD) activity was significantly increased in the 0.25FMO, 1FMO, and 2FMO groups, and the malondialdehyde (MDA) content was significantly decreased while the glutathione peroxidase (GSH-Px) content was significantly increased in the 0.5FMO, 1FMO, and 2FMO groups (*p* < 0.05). Furthermore, the 1FMO group was observed to significantly increase the abundance of Firmicutes while simultaneously reducing the abundance of *Aeromonas* (*p* < 0.05) and adjusting the structure of the intestinal microbiome. In conclusion, this study established the optimal fermentation conditions for *M. oleifera* and obtained a product with high nutrient and low tannin contents. Furthermore, the incorporation of 1% FMO was demonstrated to facilitate growth, enhance the antioxidant capacity, and optimize the gut microbiology in crayfish.

## 1. Introduction

*Moringa oleifera* is a plant of the genus *Moringa* in the Moringaceae family, which, because of its high nutritional value, such as proteins, fatty acids, and vitamins, is of interest to many experts and scientists [1]. *M. oleifera* leaves have been demonstrated to provide humans with nutrients when consumed directly or incorporated into foodstuffs [2,3,4]. For animals, the search for a cheap and nutritious alternative feed has become more problematic as feed prices continue to rise. As *M. oleifera* is rich in nutrients, the use of *M. oleifera* as a feed substitute for soybean meal, fish meal, and other feeds has become a concern for scholars at present [5,6]. Vargas-Sánchez et al. (2019) made *M. oleifera* leaves into silage for dairy cows and found that the cows had a stronger immune system than those fed normal silage [7]. At present, a lot of *M. oleifera* feed is used for ruminant and livestock animals for achieving better breeding results [8,9], but we have also found that *M. oleifera* leaves contain anti-nutritional factors, such as tannins, which can reduce the digestibility of nutrients and impair intestinal health if consumed in excess [10,11]. It is, therefore, crucial to identify an effective method for increasing the nutrient contents and reducing the anti-nutritional factors present in *M. oleifera* leaves, as this will be of great significance for the future utilization of *M. oleifera* leaves as animal feed. Numerous studies have shown that the protein contents of plant products and palatability of feeds can be improved through fermentation techniques [12,13,14]. Typically, plants are fermented to reduce the carbohydrate content of indigestible oligosaccharides and polysaccharides, which improves vitamin B availability and amino acid synthesis [15]. Zhang et al. (2017) have found that solid-state fermentation using *Bacillus subtilis* CICC 10440 mixed with *M. oleifera* leaf powder at a ratio of 1:60 produces the maximum amount of soluble protein [16].

*Procambarus clarkii*, also known as crayfish, is preferred by a wide range of consumers for its nutritious and tasty meat [17]. However, with the rapid development of aquaculture, there are more diseases and a shortage of protein-source feed resources, such as fishmeal and soybean meal, and feed prices continue to rise, affecting the development of the entire feed industry. Siddik et al. (2020) demonstrated that the replacement of fishmeal with fermented animal-source protein improved the gut microbiology, immune performance, and resistance to *Vibrio mimicus* in crayfish [18]. Jerimoth et al. (2023) showed that the addition of 1% *M. oleifera* leaves increased the growth performance of *Clarias gariepinus* [19]. Kaleo et al. (2019) showed that the addition of 0.5% *M. oleifera* leaf extracts improved the growth performance, increased immunity, and alleviated the stress caused by high ammonia levels in *Macrobrachium rosenbergii* [20]. Kamble et al. (2019) demonstrated that the addition of 0.5% *M. oleifera* leaves was able to improve the growth and immunity of Nile tilapia [21].

However, the use of fermented *M. oleifera* leaves as a functional feed for crayfish is still less researched. Therefore, these experiments were conducted to obtain the optimal fermentation conditions of *M. oleifera* leaves by liquid fermentation and to study the effects of fermented *M. oleifera* leaves as feed additives on the growth performance and antioxidant capacity of crayfish as an experimental object, thus laying the foundation for the application of fermented *M. oleifera* leaf feed in the aquaculture process.

## 2. Materials and Methods

### 2.1. M. oleifera Leaves, Probiotics, and Crayfish

*M. oleifera* leaves were obtained from Xi’an Xihai Biotechnology Co., Ltd., Xi’an, China. *B. subtilis* S1X-15, *B. subtilis* JD, *B. subtilis* ZSP, *B. subtilis* IIIA-2, and *B. amyloliquefaciens* JY-24 were obtained from Jiangsu Su Wei Microbiology Research Co., Ltd., Wuxi, China and kept at the Freshwater Fisheries Research Centre (FFRC). The crayfish were obtained from Yangzhong Experimental Base at the Jiangsu Institute of Freshwater Aquatic Research (Zhenjiang, China).

### 2.2. Exploration of the Optimal Fermentation Conditions of M. oleifera Leaves

#### 2.2.1. Preparation of Bacterial Suspension and Fermented *M. oleifera* Leaves

The fermentation strains were inoculated in a Luria–Bertani slant medium and cultured at 37 °C for 24 h. The strains were inoculated in 3 Erlenmeyer flasks containing 15 mL of sterilized distilled water, and the concentration of the bacterial suspension was adjusted to 10^7^ CFU mL^−1^.

The dried *M. oleifera* leaves were crushed and sieved through an 80-mesh sieve to obtain powder, which was thoroughly mixed according to a material/water ratio of 2.5–12.5% to obtain pulp. The bacterial suspension was added to the fermentation tank containing the pulp at 2%, and the *M. oleifera* leaf pulp was used for the next single-factor and orthogonal experiments.

#### 2.2.2. Single-Factor Fermentation Test

##### The Optimal Material/Water Ratio of the Fermented *M. oleifera* Leaves

To determine the appropriate fermentation material/water ratio, the *M. oleifera* leaf powder was combined with water at concentrations of 2.5%, 5%, 7.5%, 10%, and 12.5% (*m*/*v*); adjusted to pH 8.6; sterilized in an autoclave at 121 °C for 30 min; and inoculated with 2% *B. subtilis* S1X-15 at a temperature of 35 °C and a shaker speed of 180 rpm. After fermentation, the sample was centrifuged for 10 min at 6000 rpm and 4 °C, and the supernatant was used to assay the total protein and total phenolic contents. Three replicates were performed for each experiment.

##### The Optimal Fermentation Temperature of the Fermented *M. oleifera* Leaves

To explore the appropriate fermentation temperatures, the fermentation temperature was set at 25 °C, 30 °C, 35 °C, 40 °C, and 45 °C. *M. oleifera* leaf powder and water were mixed at 10%, autoclaved, and then inoculated with 2% *B. subtilis* S1X-15, maintaining the shaker speed at 180 rpm for 48 h. Samples were processed and screened under the same conditions as those described in “The Optimal Material/Water Ratio of the Fermented *M. oleifera* Leaves” of Section 2.2.2. Three replicates were performed for each experiment.

##### The Optimal Fermentation Strains of the Fermented *M. oleifera* Leaves

To investigate the appropriate fermentation strains, a temperature of 35 °C, a fermentation time of 48 h, and a material/water ratio of 10% were maintained; fermentation broth samples were inoculated with *B. subtilis* S1X-15, *B. subtilis* JD, *B. subtilis* ZSP, *B. subtilis* IIIA-2, and *B. amyloliquefaciens* JY-24 at an inoculum amount of 2%; and a shaking speed of 180 rpm was maintained. Samples were processed and screened under the same conditions as those described in “The Optimal Material/Water Ratio of the Fermented *M. oleifera* Leaves” of Section 2.2.2. Three replicates were performed for each experiment.

##### The Optimal Fermentation Time of the Fermented *M. oleifera* Leaves

To obtain the appropriate fermentation time, fermentation times of 12 h, 24 h, 38 h, 72 h, and 96 h were set; the temperature was maintained at 35 °C; the material/water ratio was 10%; the samples were inoculated with 2% *B. subtilis* S1X-15; and the shaking speed was 180 rpm. Samples were processed and screened under the same conditions as those described in “The Optimal Material/Water Ratio of the Fermented *M. oleifera* Leaves” of Section 2.2.2. Three replicates were performed for each experiment.

#### 2.2.3. Orthogonal Experiment of Fermented *M. oleifera* Leaves

The orthogonal test was performed based on the one-way test according to the L_9_(3^4^) orthogonal table [22]. The total protein content was used as a screening indicator to determine the optimal conditions for *M. oleifera* leaves. The factor level and orthogonal test table are shown in Table 1. There were 3 replications for each experiment.

#### 2.2.4. Determination of the Total Protein Content

The total protein content was determined according to the experimental method proposed by Loffler [23]. A bovine serum albumin protein standard (the protein was obtained from Beijing Solarbio Technology Co., Ltd., Beijing, China; kit code: A8020) was diluted twofold, and the enzyme assay was carried out at a wavelength of 595 nm. A standard curve was constructed, and the concentration of the sample protein was calculated according to the protein standard curve.

#### 2.2.5. Determination of the Flavonoid Content

The methodology was followed according to the description of the plant flavonoid content test kit from Beijing Solarbio Technology Co., Ltd. (kit code: BC1335). Samples were extracted by ultrasonic extraction with 60% ethanol at 60 °C for 30 min. The tannic acid standard was diluted twice, and the enzyme standard was measured at 470 nm to construct a standard curve according to the tannic acid standard curve to calculate the content of flavonoids in the sample.

#### 2.2.6. Determination of the Total Phenol Content

The test kit for the total phenol content from Beijing Solarbio Technology Co., Ltd. (kit code: BC1345) was employed. The standard solution of tannic acid was diluted twofold, and the enzyme assay was performed at a wavelength of 760 nm to construct a standard curve. The content of the total phenol in the samples was then calculated according to the standard curve.

#### 2.2.7. Determination of the Tannin Content

The tannin content was checked using the test kit of Beijing Solarbio Technology Co., Ltd. (kit code: BC1395). Briefly, the samples were extracted with the extract solution and then subjected to a water bath at 70 °C for 30 min. The tannin standard solution was diluted twice with the extract solution and then subjected to an enzymatic assay at 275 nm to construct a standard curve, which was then used to calculate the tannin content in the samples.

#### 2.2.8. Determination of the Total Amino Acid Content

In accordance with the specifications set forth by Nanjing Jiancheng Biotechnology Co., Ltd., Nanjing, China (kit code: A026-1-1), the total amino acid determination kit is to be utilized. A volume of 500 µL of the supernatant was transferred to a 2 mL centrifuge tube, followed by the addition of 1 mL of the amino acid reaction solution. Subsequently, the amino acid developing solution was added, and the mixture was mixed. The enzyme assay was then conducted at a wavelength of 650 nm, according to the standard curve, in order to calculate the content of the total amino acids in the samples.

### 2.3. Experimental Culture of Crayfish

#### 2.3.1. Experimental Design and Diet

*M. oleifera* leaves were fermented under the optimal conditions, and the liquid was pre-cooled at −80 °C for 2 h and freeze dried at −50 °C for 48 h to obtain the fermented *M. oleifera* leaf powder. The culture experiment was designed as a completely randomized experimental design, with 450 healthy and similarly sized crayfish (9.11 ± 0.3 g), which were then divided into five groups with 0% (CT), 0.25% (0.25FMO), 0.5% (0.5FMO), 1% (1FMO), and 2% (2FMO) doses of fermented *M. oleifera* leaves. Each group consisted of 30 crayfish with three replicates and was cultured in fifteen cement ponds (600 L; 1 m × 1m × 0.6 m) for 60 days.

The formulations and approximate compositions of the experimental diets are shown in Table 2. Various raw materials were crushed through a 60-mesh sieve and gradually mixed and then oil and an appropriate amount of water were added. A twin-screw extruder, manufactured by Guangzhou Huagong Optical, Mechanical & Electrical Technology Co., Ltd. in Guangzhou, China, was used for granulating the sinking pellets.

#### 2.3.2. Feeding Management

In order to provide an optimal environment for the crayfish, tubes and tiles were introduced to each pond. Following a three-day cultivation period, the crayfish were provided with sustenance on three occasions per day, at 7:00, 12:00, and 19:00. Adaptations were made according to the dietary requirements of the crayfish. The aquaculture process was maintained at a depth of 30 cm, with waterflow maintained at 1 L min^−1^ within the recirculation system. Rearing conditions included a temperature of 29–30 °C, a pH range of 7.0–7.5, and dissolved oxygen levels above 5 mg L^−1^, with ammonia, nitrogen, and nitrite levels below 0.1 mg L^−1^.

#### 2.3.3. Sample Collection and Growth Indicator Determination

Following an extensive 60-day breeding period, the crayfish underwent a 24-h fasting phase. The total weight and dietary intake were measured, yielding data on the weight-gain rate and feed conversion rate. Hepatopancreatic and intestinal tissues (6 per tank, 18 per group) were preserved at −20 °C for antioxidant and digestive enzyme assessments. Another crayfish from the same tank had their intestines promptly harvested and stored at −80 °C for subsequent gut microbiome analyses.

The growth rate (WGR), specific weight-gain rate (SGR), and feed conversion ratio (FCR) for the crayfish were calculated as follows:

                             WGR (%) = (G_2_ − G_1_)/G_1_ × 100

                                     SGR (%/d) = (lnG_2_ − lnG_1_)/D × 100

FCR = G_3_/G′

Notes: G_1_ is the average initial weight of the crayfish (g), G_2_ is the average final weight of the crayfish (g), D is the duration of the culture trial (days), G_3_ is the total food intake (g), G′ is the total weight gain.

#### 2.3.4. Determination of Digestive Enzymatic Activities

The intestines of the crayfish were used for the determination of the α-amylase, lipase, and trypsin activities. Digestive enzyme kits were purchased from Nanjing Jiancheng Biotechnology Co., Ltd. (α-amylase kit code: C016-1-1, lipase kit code: A054-1-1, and trypsin kit code: A080-2-2).

#### 2.3.5. Determination of the Antioxidant Enzymatic Activities

For the determination of the antioxidant capacities, the hepatopancreases of the crayfish and the total antioxidant capacity (kit code: A015-1-2), total superoxide dismutase (kit code: A001-1), malondialdehyde (kit code: A003-1), glutathione peroxidase (kit code: A005-1-2), and catalase (kit code: A007-1-1) test kits from Nanjing Jiancheng Biotechnology Co., Ltd. were used.

The methods for the determination of the glutamic transaminase (AST) and glutamic pyruvic transaminase (ALT) activities were based on the kits supplied by Nanjing Jiancheng Biotechnology Co., Ltd. The kits used were the AST kit (code: C010-1-1) and the ALT kit (code: C009-1-1).

#### 2.3.6. Microbiome Analysis

The intestines of the CT, 1FMO, and 2FMO groups were transported via dry ice to Nanjing Genepioneer Biotechnologies Co., Ltd., Nanjing, China, with 3 replicates in each group. DNA was extracted with an E.Z.N.A.^®^ Soil Kit (Omega Bio-Tek, Norcross, GA, USA), and primers were designed to amplify the V3-V4 region to yield a 420 bp fragment. The Illumina NovaSeq 6000 platform (Illumina, San Diego, CA, USA) was used to sequence the data. The Silva 16S rRNA database was selected for the 16S rRNA motif. By comparing amplicon sequence variant (ASV) sequences to those in the Silva bacterial database, the species classification information corresponding to each ASV was obtained, and the most abundant sequences were selected. The QIIME2 consensus research algorithm was used for classifying each ASV feature sequence, and species annotation was performed using default parameters in QIIME2 software (qiime2-2021.11).

#### 2.3.7. Bioinformatic Analysis

The annotated species were employed for microbiological analyses, including the diversity index, cluster analysis, differential analysis, KEGG pathway prediction, and correlational analysis. The diversity index was compared between the groups by analyzing the observed species, the Chao1 index, and the Shannon and Simpson indices (*p* < 0.05 indicated a significant difference). The magnitude of the abundance was observed by clustering species at the genus and phylum levels (*p* < 0.05 indicated a statistically significant difference in the abundance). The differential species for each group were obtained by LEfSe analysis (*p* = 0.05; LDA = 4). The KEGG pathways in each group were obtained by comparing KEGG libraries, including KEGG primary and secondary pathways. Correlational analyses of phylum- and genus-level species with antioxidant indicators were performed in order to observe the levels of the associations between gut microbes and antioxidant indicators [24].

### 2.4. Statistical Analysis

The data were analyzed by analysis of variance (ANOVA) and multiple comparisons (Duncan) using SPSS 20.0 software. An independent sample *t*-test was performed to test the differences before and after the fermentation of the *M. oleifera* leaves. The results are expressed as means ± SDs. *p* < 0.05 was considered to indicate a significant difference. Bioinformatic analysis was performed using OmicStudio tools at https://www.omicstudio.cn/tool (accessed on 1 April 2024).

## 3. Results

### 3.1. Exploration of the Optimal Fermentation Conditions of M. oleifera Leaves

#### 3.1.1. The Optimal Material/Water Ratio of the Fermented *M. oleifera* Leaves

**Figure 1:**
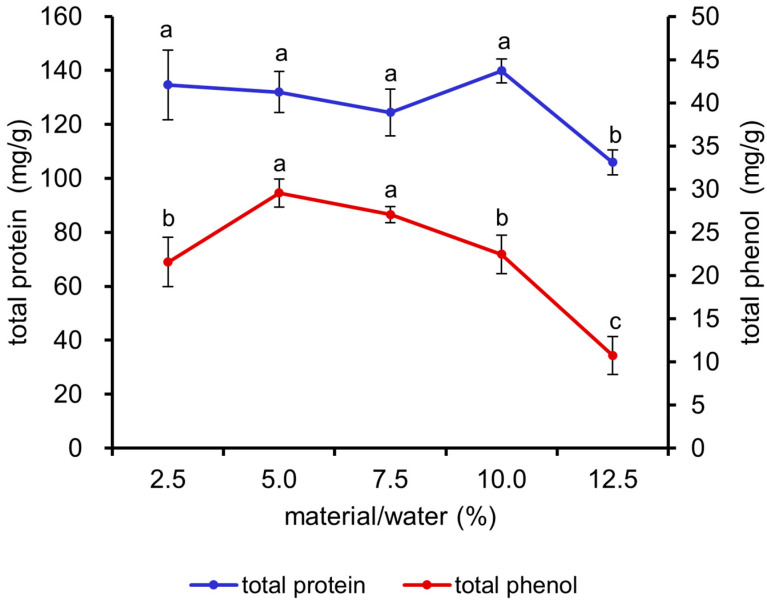
Effects of different material/water ratios on the total protein and total phenol contents of fermented *M. oleifera* leaves. Note: different letters in the figure indicate significant differences, *p* < 0.05.

As illustrated in Figure 1, the total protein content exhibited a significant decrease at a 12.5% material/water ratio (*p* < 0.05). The total phenol content demonstrated an initial increase, followed by a decline, with increasing material/water ratio, and the maximum value was attained at ratios of 5% and 7.5% (*p* < 0.05). Consequently, the 5%, 7.5%, and 10% ratios were selected for the orthogonal experiment.

#### 3.1.2. The Optimal Fermentation Temperature of the Fermented *M. oleifera* Leaves

**Figure 2:**
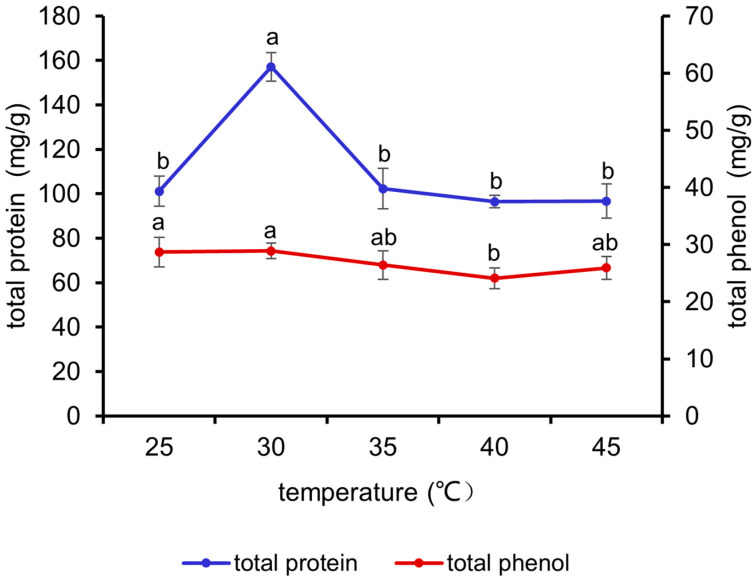
Effects of different temperatures on the total protein and total phenol contents of fermented *M. oleifera* leaves. Note: different letters in the figure indicate significant differences, *p* < 0.05.

It can be seen from Figure 2 that the total protein content exhibited an initial increase, followed by a decline, with increasing fermentation temperature and reached the maximum value at 30 °C (*p* < 0.05). The total phenol content demonstrated a decline with increasing temperature. Accordingly, 25 °C, 30 °C, and 35 °C were selected for the orthogonal experiment.

#### 3.1.3. The Optimal Fermentation Strains of the Fermented *M. oleifera* Leaves

**Figure 3:**
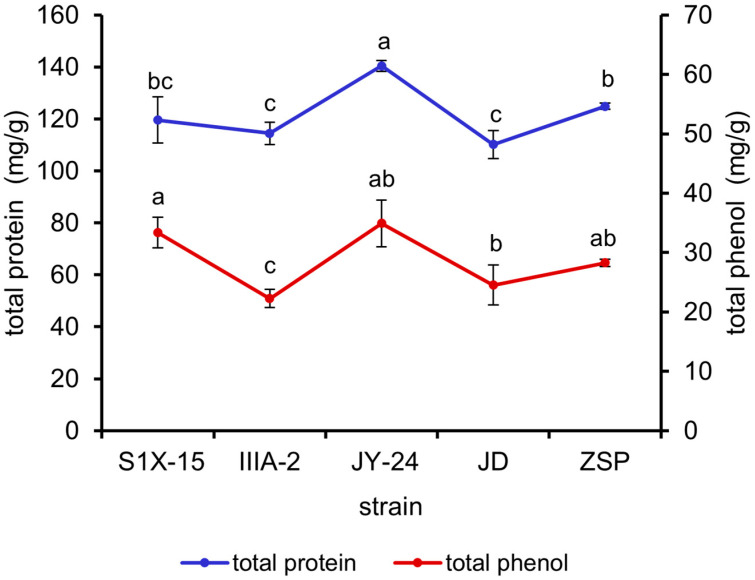
Effects of different strains on the total protein and total phenol contents of fermented *M. oleifera* leaves. Note: different letters in the figure indicate significant differences, *p* < 0.05.

The results in Figure 3 reveal that the total protein content of the *M. oleifera* leaves fermented by *B. amyloliquefaciens* JY-24 was significantly higher than those of the *M. oleifera* leaves fermented by the other strains (*p* < 0.05). The total phenol contents of the *B. subtilis*-S1X-15- and *B. amyloliquefaciens*-JY-24-fermented *M. oleifera* leaves were found to be higher than those of the *M. oleifera* leaves fermented by the remaining strains. Therefore, *B. subtilis* S1X-15, *B. subtilis* IIIA-2, and *B. amyloliquefaciens* were selected for orthogonal experiments.

#### 3.1.4. The Optimal Fermentation Time of the Fermented *M. oleifera* Leaves

**Figure 4:**
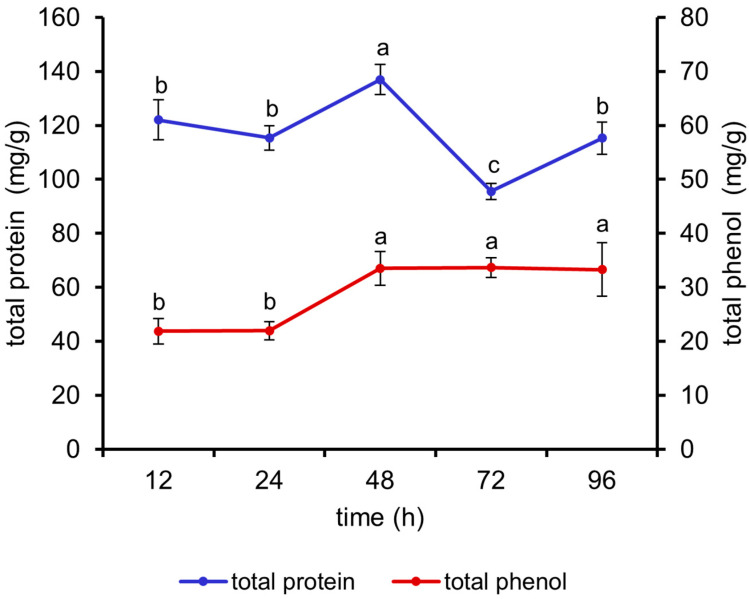
Effects of different fermentation times on the total protein and total phenol contents of fermented *M. oleifera* leaves. Note: different letters in the figure indicate significant differences, *p* < 0.05.

As can be seen in Figure 4, the total protein content of the fermented *M. oleifera* leaves reached the maximum value at 48 h (*p* < 0.05). The total phenol content increased with increasing fermentation time and started to increase significantly at 48 h (*p* < 0.05), with no significant difference between 48 and 96 h. Therefore, 24 h, 48 h, and 72 h were selected for orthogonal experiments.

#### 3.1.5. Orthogonal Experiment of Fermented *M. oleifera* Leaves

The results of the single-factor experiment were used to design the orthogonal tests, which were carried out to determine the most appropriate fermentation conditions. The results of the extreme difference analysis are presented in Table 3, with the total protein content used as an indicator. The primary relationship among the factors influencing the fermentation conditions was C > A > B > D. The optimal combination was identified as C_1_ A_3_ B_2_ D_2_, which corresponds to the fermentation strain *B. amyloliquefaciens* JY-24, a material/water ratio of 10%, a fermentation time of 48 hours, and a fermentation temperature of 30 °C.

### 3.2. Changes in Nutrient Composition and Tannin Content of Fermented M. oleifera Leaves

The results before and after fermentation for *M. oleifera* leaves are illustrated in Table 4. Under the optimal conditions, the contents of the total protein, total phenolics, flavonoids, and amino acids before fermentation exhibited a marked increase (*p* < 0.01), while the content of tannins was significantly reduced in comparison to that observed after fermentation (*p* < 0.05).

### 3.3. Effect of Fermented M. oleifera Leaves on the Growth Performance of the Crayfish

The results of the growth performance of the crayfish are shown in Table 5. FBW, WGR, and SGR were significantly higher in the 1FMO group than those of the control and 0.25FMO groups (*p* < 0.05), and the difference in FCR among the groups was not significant (*p* > 0.05).

### 3.4. Effect of Fermented M. oleifera Leaves on the Digestive Enzymes of the Crayfish

**Figure 5:**
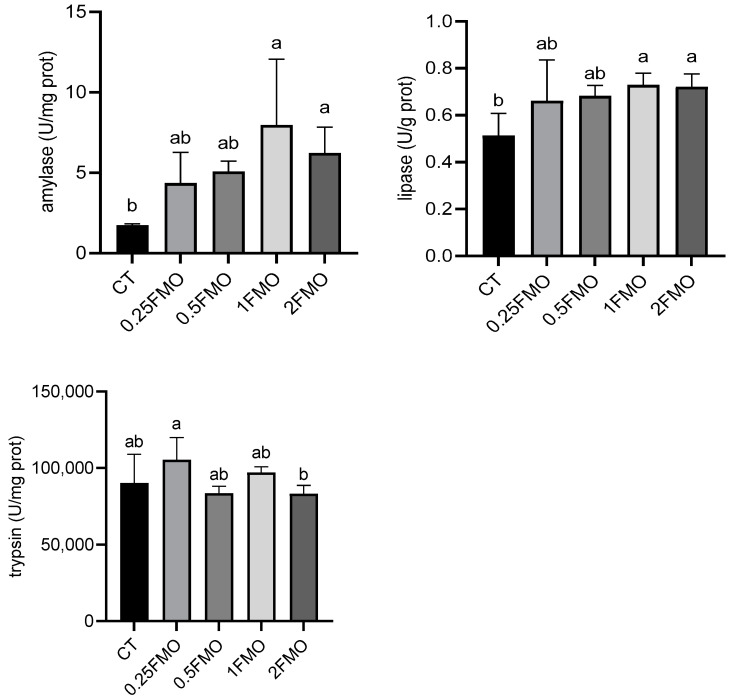
Effect of fermented *M. oleifera* leaves on the digestive enzymes of the crayfish. Note: different letters in the different groups indicate significant differences, *p* < 0.05.

The results of digestive enzymatic activities are shown in Figure 5. The addition of fermented *M. oleifera* leaves significantly increased the α-amylase activity (*p* < 0.05). The 1FMO and 2FMO groups showed significantly increased lipase activity (*p* < 0.05), and the 2FMO group showed significantly decreased trypsin activity compared to the 0.25 group (*p* < 0.05), while the rest of the additive groups did not show any significant difference compared to the control group (*p* > 0.05).

### 3.5. Effect of Fermented M. oleifera Leaves on the Antioxidant Capacity of the Crayfish

**Figure 6:**
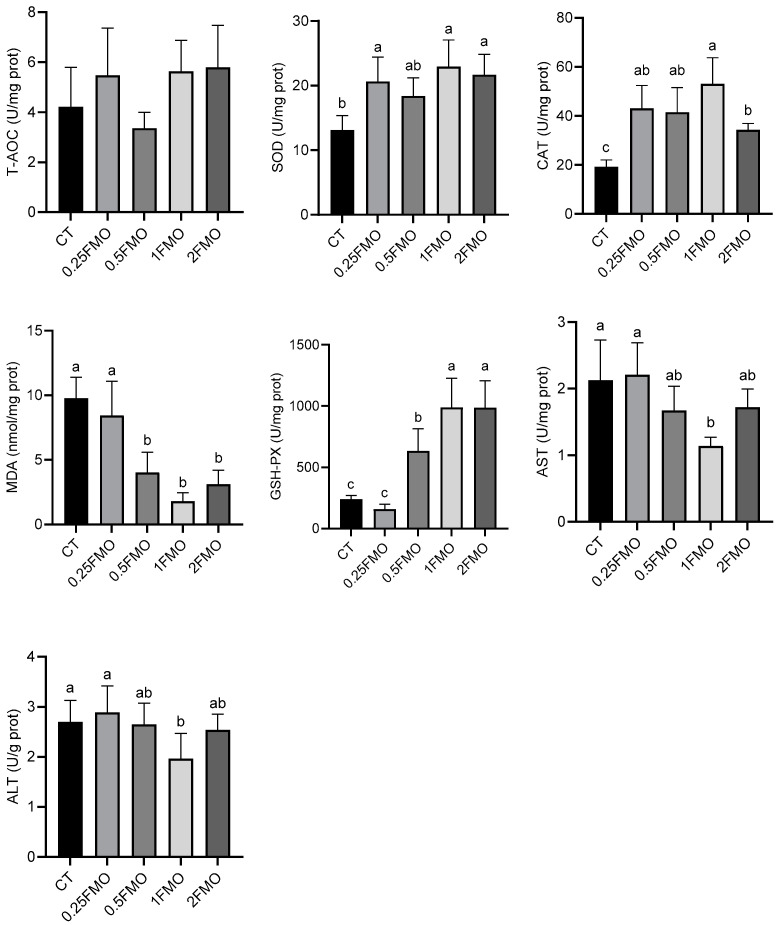
Effect of fermented *M. oleifera* leaves on the antioxidant capacity of the crayfish. Note: different letters in the different groups indicate significant differences, *p* < 0.05.

The results of the antioxidant enzymatic activities of the crayfish are shown in Figure 6. There was no significant difference in the total antioxidant capacity (T-AOC) among the groups (*p* > 0.05). The 0.25FMO, 1FMO, and 2FMO groups showed significantly increased SOD activity in contrast with the CT group (*p* < 0.05); the addition of the fermented *M. oleifera* leaves significantly increased the CAT activity compared to that of the CT group (*p* < 0.05). The 0.5FMO, 1FMO, and 2FMO groups showed significantly decreased MDA contents and increased GSH-Px activities compared to those of the CT group (*p* < 0.05), and the 1FMO group showed significantly reduced AST and ALT contents in contrast to those of the CT group (*p* < 0.05).

### 3.6. Effect of Fermented M. oleifera Leaves on the Intestinal Microbiome of the Crayfish

#### 3.6.1. Diversity Analysis of Intestinal Communities

**Figure 7:**
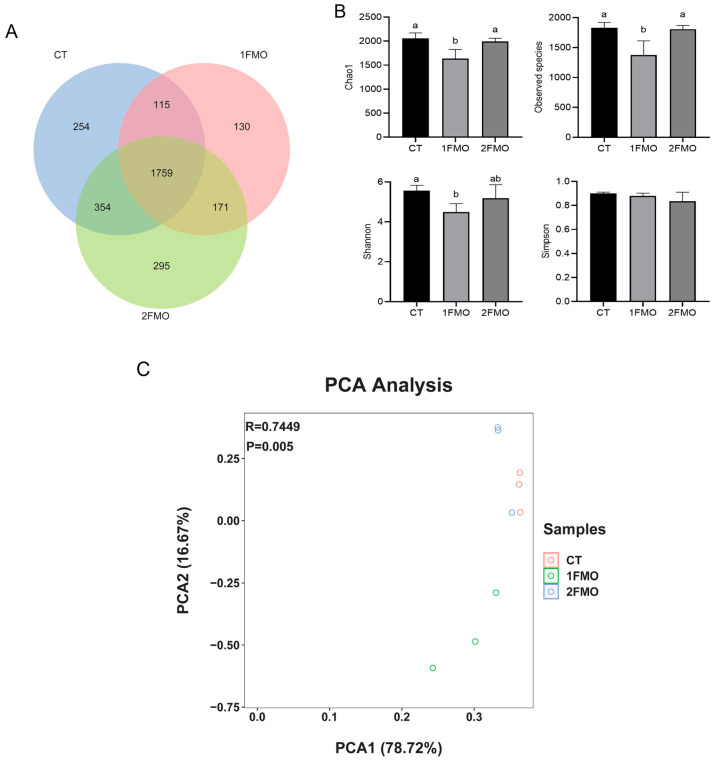
Effect of fermented *M. oleifera* leaves on the diversity analysis of the intestinal communities of the crayfish. Note: different letters in the different groups indicate significant differences, *p* < 0.05. (**A**): Venn diagram; (**B**): α-diversity; (**C**): PCA analysis.

A Venn diagram was constructed based on the cluster analysis of the OTUs (Figure 7A). The number of OTUs shared by the CT, 1FMO, and 2FMO groups was 1759, of which the number of OTUs specific to the 2FMO group was 295; the number of OTUs specific to the CT group was 254, and the number of OTUs specific to the 1FMO group was 130.

After 60 days of culturing, the results of the crayfishes’ gut microbiome diversity are shown in Figure 7B. Chao1 and the observed species index were significantly lower in the 1FMO group than those of the CT and 2FMO groups (*p* < 0.05); the Shannon index of the 1FMO group was significantly lower than that of the CT group (*p* < 0.05), and there was no significant difference in the Simpson index between the groups (*p* > 0.05).

Analysis of the PCA results showed that the differences in the community structure between the CT and 2FMO groups were small, and the differences in the community structure between the 1FMO and CT groups were large (Figure 7C).

#### 3.6.2. Analysis of the Structure of the Intestinal Microbiome of the Crayfish

**Figure 8:**
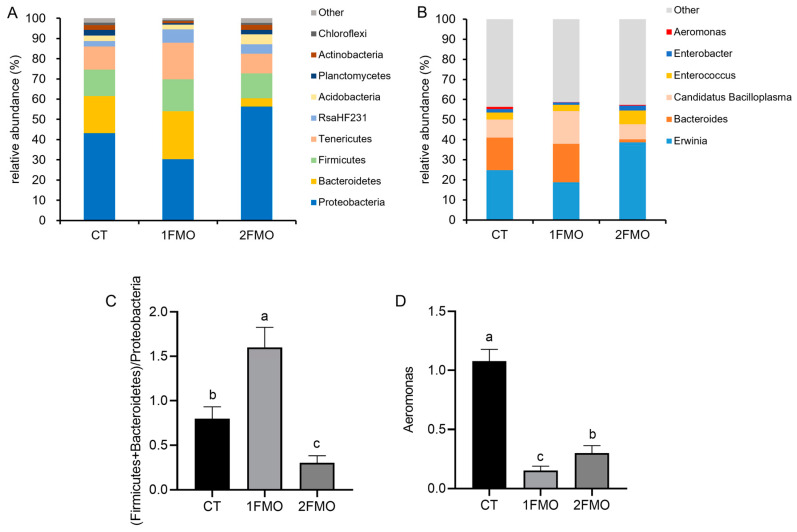
Effect of fermented *M. oleifera* leaves on the intestinal microbiome of the crayfish. Note: different letters in the different groups indicate significant differences, *p* < 0.05. (**A**): phylum level; (**B**): genus level; (**C**): the ratio of (Firmicutes + Bacteroidetes)/Proteobacteria; (**D**): the abundance of *Aeromonas*.

At the phylum level, Proteobacteria, Bacteroidetes, Firmicutes, and Tenericutes were the major phyla in the three groups (Figure 8A); at the genus level, *Erwinia*, *Bacteroides*, *Candidatus Bacilloplasma*, and *Enterococcus* were the major genera in the three groups (Figure 8B).

The (Firmicutes + Bacteroidetes)/Proteobacteria ratio was significantly higher in the 1FMO group than in the CT group, while the ratio was significantly lower in the 2FMO group (*p* < 0.05, Figure 8C); the abundance of *Aeromonas* was significantly lower in the 1FMO group than in the CT and 2FMO groups (*p* < 0.05, Figure 8D).

#### 3.6.3. Differential Analysis of the Intestinal Microbiome of the Crayfish

**Figure 9:**
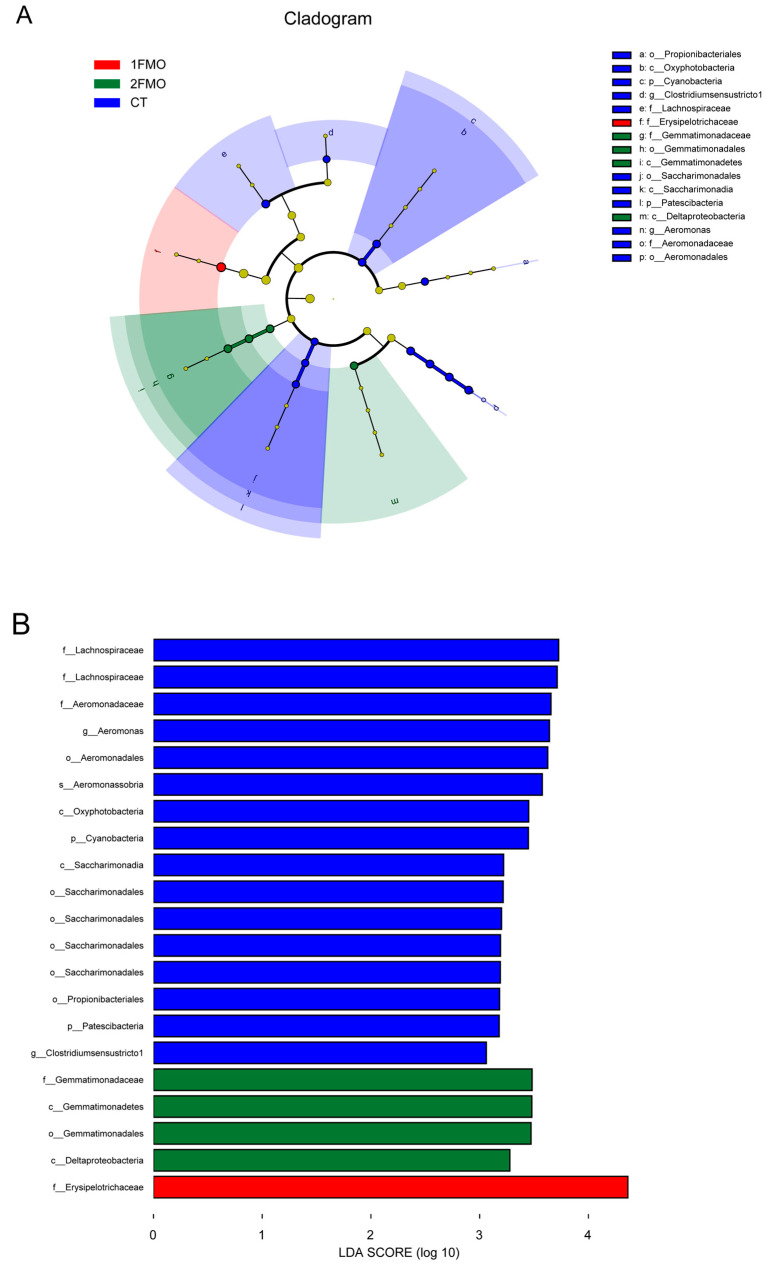
Differential analysis of the effect of fermented *M. oleifera* leaves on the intestinal microbiome of the crayfish. Note: (**A**): species evolutionary branching diagram; (**B**): histogram of LDA distribution.

The LEfSe analysis results are shown in Figure 9 (*p* = 0.05, LDA = 3). The main differentiating species in the CT group were the species *Aeromonas sobria* of the phylum Proteobacteria, the order Saccharimonadales of the phylum Patescibacteria, the family Lachnospiraceae and genus *Clostridium sensu stricto* of the phylum Firmicutes, the class Oxyphotobacteria of the phylum Cyanobacteria, and the order Propionibacteriales of the phylum Actinobacteria. The main differential species in the 1FMO group was the family Erysipelotrichaceae of the phylum Firmicutes. The main differential species in the 2FMO group were the family Gemmatimonadaceae of the phylum Gemmatimonadetes and the class Deltaproteobacteria of the phylum Proteobacteria.

#### 3.6.4. KEGG Analysis

**Figure 10:**
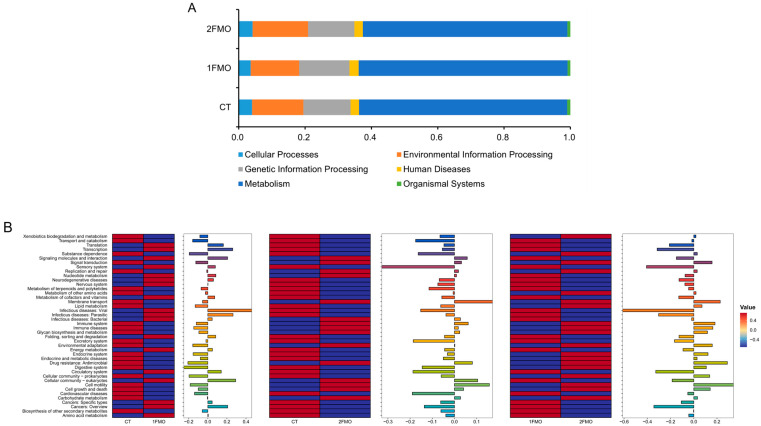
KEGG analysis of the effect of fermented *M. oleifera* leaves on the crayfish. Note: (**A**): KEGG Level-1 pathway; (**B**): KEGG Level-2 pathway.

The results of the KEGG-enriched pathway analysis are shown in Figure 10. The CT group, 1FMO group, and 2FMO group pathways were mainly enriched in cellular processes, genetic information processes, metabolism processes, environmental information processes, human diseases, and organismal systems, especially metabolic systems.

The results of the KEGG secondary enrichment pathway showed that the 1FMO group increased the expressions of Signaling molecules and the interaction, Cancers: Overview, Transcription, Infectious diseases: Parasitic, Cellular community—eukaryotes and Infectious diseases: Viral pathways; and decreased the expression of Digestive system and Drug resistance: Antimicrobial pathways compared to the CT group (|log_2_FC| > 0.2). The 2FMO group decreased the expression of the Sensory system pathway compared to those of the CT group (|log_2_FC| > 0.2). Compared with the 2FMO group, the 1FMO group increased the expressions of Infectious diseases: Viral, Sensory system, Cancers: Overview, Circulatory system, Transcription, and Infectious diseases: Parasitic pathways; and decreased the expressions of the Membrane transport, Drug resistance: Antimicrobial and Cell motility pathways (|log_2_FC| > 0.2).

### 3.7. Correlational Analysis of Microbial Genera with Antioxidant Indices

**Figure 11:**
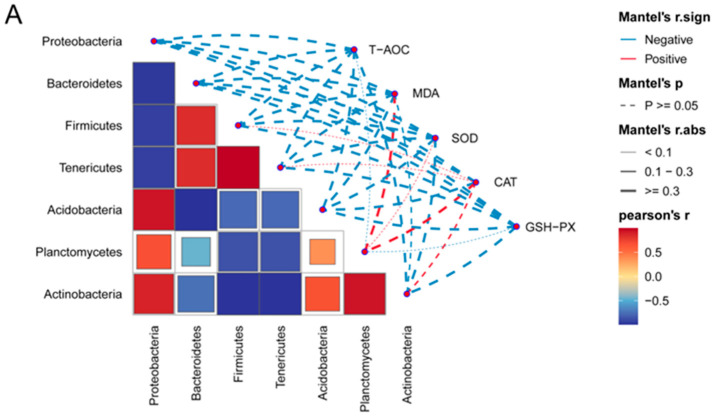

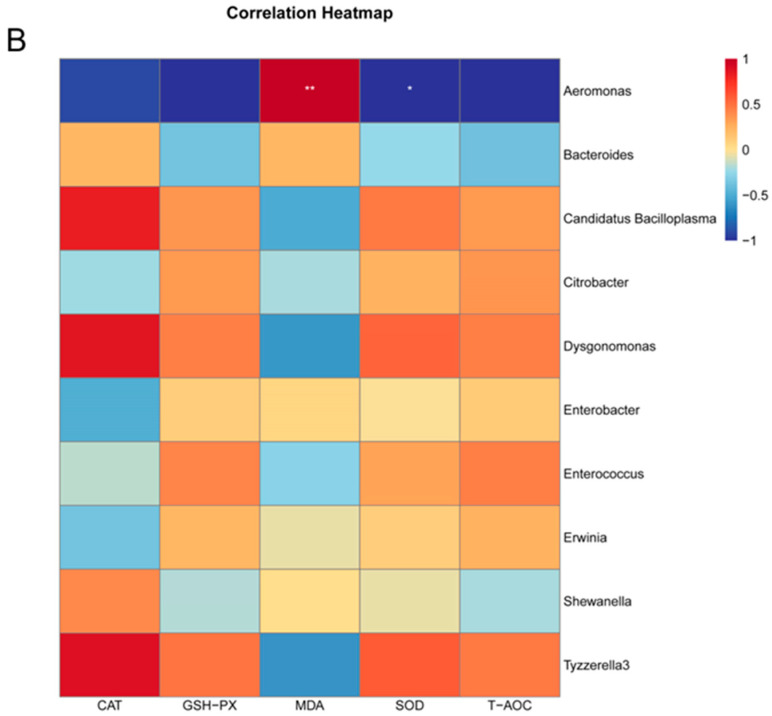
Correlational analysis of microbial genera with antioxidant indices. Note: (**A**): phylum level; (**B**): genus level. “*” represents significant differences, and “**” represents extremely significant differences.

The crayfish gut phylum-level microorganisms and genus-level microorganisms were correlated with antioxidant indicators, and the results are shown in Figure 11. At the phylum level (Figure 11A), T-AOC and GSH-Px were all negatively correlated with Proteobacteria, Bacteroidetes, Firmicutes, Tenericutes, Acidobacteria, Planctomycetes, and Actinobacteria (*p* > 0.05). MDA and SOD were negatively correlated with Proteobacteria, Bacteroidetes, Firmicutes, Tenericutes, Acidobacteria, and Actinobacteria and positively correlated with Planctomycetes (*p* > 0.05). CAT was negatively correlated with Proteobacteria, Bacteroidetes, and Acidobacteria and positively correlated with Firmicutes, Tenericutes, Planctomycetes, and Actinobacteria (*p* > 0.05).

At the genus level (Figure 11B), T-AOC was negatively correlated with *Aeromonas*, *Bacteroides*, and *Shewanella* and positively correlated with *Tyzzerella3*, *Enterococcus*, *Dysgonomonas*, *Citrobacter*, *Candidatus Bacilloplasma*, *Erwinia*, and *Enterobacter* (*p* > 0.05). SOD was positively correlated with *Tyzzerella3*, *Dysgonomonas*, *Candidatus Bacilloplasma*, *Enterococcus*, *Citrobacter*, and *Erwinia*; negatively correlated with *Bacteroides*, *Shewanella*, and *Enterobacter* (*p >* 0.05); and significantly negatively correlated with *Aeromonas* (*p* < 0.05). MDA was negatively correlated with *Tyzzerella3*, *Dysgonomonas*, *Candidatus Bacilloplasma*, *Enterococcus*, *Citrobacter*, and *Erwinia*; positively correlated with *Bacteroides*, *Shewanella*, and *Enterobacter* (*p >* 0.05); and significantly positively correlated with *Aeromonas* (*p* < 0.05). GSH-Px was positively correlated with *Tyzzerella3*, *Dysgonomonas*, *Candidatus Bacilloplasma*, *Enterococcus*, *Citrobacter*, *Erwinia*, and *Enterobacter* and negatively correlated with *Aeromonas*, *Bacteroides*, and *Shewanella* (*p >* 0.05). CAT was positively correlated with *Tyzzerella3*, *Dysgonomonas*, *Candidatus Bacilloplasma*, *Shewanella*, and *Bacteroides* and negatively correlated with *Aeromonas*, *Enterobacter*, *Erwinia*, *Citrobacter*, and *Enterococcus* (*p >* 0.05).

## 4. Discussion

So far, we have found that fermentation can reduce anti-nutrient factors and improve the utilization of *M. oleifera* leaves. The main factors affecting fermentation are the strains, pH, material/water ratio, strain inoculation concentration, temperature, and time [13]. Protein and polyphenols are the main nutrients in *M. oleifera* leaves and can be a good source of antioxidants [25]; therefore, they were selected as screening indicators. Fermentation time is a key factor affecting the final fermentation product, and, typically, the rate of fermentation product production slows, or even stagnates, as the fermentation time increases. Too long or too short of a fermentation time can lead to increased costs or reduced fermentation quality [26]. In this experiment, the results showed that the protein and total phenolic contents were the highest when the fermentation time was 48 h. Ali et al. (2020) proved that the total amino acid and total isoflavone contents were the highest in the fermented *M. oleifera* leaves at 48 h, and it tended to decrease when the fermentation time reached 72 h and 96 h. These results proved that the nutrient content in the fermentation broth did not increase with increasing fermentation time [27]. Numerous studies have shown that fermentation strains have an important influence on fermentation products, and the selection of strains directly affects the quality of fermentation products [28,29]. In aquaculture, *Bacillus* is the most studied host-related bacteria, which is mainly because of the unique ability of *Bacillus* to grow in the intestinal tracts of aquatic animals. In addition, *Bacillus* can secrete a variety of digestive enzymes and keep the intestinal tract in an anaerobic environment, which can effectively inhibit the growth of pathogenic bacteria and improve the immunity of aquatic animals and their resistance to pathogenic bacteria [30]. In this study, the fermentation effect of *B. amyloliquefaciens* JY-24 was better than those of *B. subtilis* S1X-15, *B. subtilis* JD, and *B. subtilis* ZSP. Temperature is another important factor affecting the fermentation product, and microbial organisms can only deliver the maximum benefit at the optimal temperature. A fermentation temperature that is too high will lead to microbial organisms’ protein denaturation, reducing the rates of microbial growth and reproduction. On the contrary, if the fermentation temperature is too low, it will inhibit microbial organisms’ production of enzymes and reduce their enzymatic activities [31]. In this work, the total phenolic and protein contents appeared to increase and then decrease with increasing temperature, and the contents reached the highest at 30 °C. Therefore, 30 °C was selected as the optimal fermentation temperature. Water is the basis for the growth and reproduction of microorganisms and is necessary to assist microorganisms in their metabolism, absorption, and secretion of nutrients and in other life activities. The quality of the fermented feed is affected by the level of the water content, which, if too high or too low, will lead to contamination by stray bacteria or decreased nutrient contents [32]. In this study, the protein and total phenolic contents showed a tendency to increase and then decrease with increasing material/water ratio, and the appropriate range was between 5% and 10%.

Based on the single-factor results and L_9_(3^4^) orthogonal test results, the optimal fermentation conditions of *M. oleifera* were the fermentation strain *B. amyloliquefaciens* JY-24, a fermentation material/water ratio of 10%, a fermentation time of 48 h, and a fermentation temperature of 30 °C. Large molecules were broken down into organic acids, soluble peptides, and other small molecules when plants were fermented, increasing the nutrient content and reducing the levels of harmful substances [33]. From the experimental results, it was observed that the protein content, amino acid content, total phenolic content, and flavonoid content of the fermented *M. oleifera* leaves were increased by 24.53%, 36.21%, 82.81%, and 58.58%, respectively, as compared to those of the unfermented leaves. The tannin content of the *M. oleifera* leaves was reduced by 8.4%. Tannins are the major anti-nutritional factor in *M. oleifera* leaves. Excessive levels of tannins in feeds affect palatability and reduce feed intake, and tannins are biodegraded in the gastrointestinal tract to produce low-molecular-weight phenolic compounds (which have a direct toxic effect on food animals), as well as metal ions, enzymes, sugars, and vitamin B12, and reduce feed utilization [34].

Studies have shown that during the fermentation process, probiotics ‘pre-digest’ the fermentation substrate by breaking down anti-nutritional factors and releasing nutrients [35,36]. Fermented feeds can form ‘biological, nutritional, and chemical barriers’ to promote growth, improve immunity, and protect gut health after feeding in aquatic animals [37,38]. Das et al. (2022) showed that solid-state fermentation of hemp oil cake by mixed strains (*Saccharomyces cerevisiae* and *B. subtilis*) increased its crude protein content and reduced crude fiber and anti-nutritional factors, such as tannins and saponins, and that replacing 20% of the soybean meal in the diet with fermented hemp oil cake significantly increased the growth performance, feed conversion ratio, and nutrient digestibility of *Labeo rohita* [39]. In this study, the addition of 1% fermented *M. oleifera* leaves to the feed improved the growth performance and feed utilization of the crayfish compared to the control group. The intestinal tract is the main place for the digestion and absorption of nutrients in aquatic animals, and the activities of digestive enzymes in crayfish highly reflect their ability to obtain nutrients from food, as well as their growth and development [40]. Dai et al. (2017) found that the digestive enzymatic activities of fast-growing shrimp were significantly higher than those of slow-growing shrimp and that the digestive enzymatic activities were positively correlated with the shrimps’ body weight and body length [41]. In the present study, the intestinal digestive enzymatic activities of the crayfish were significantly higher in the 1FMO and 2FMO groups than in the group without the addition of the fermented *M. oleifera* leaves. It was found that the addition of the *L. acidophilus*- and *S. cerevisiae*–solid-fermented seaweed (*Enteromorpha prolifera*) to the diet at 20–50 g kg^−1^ significantly increased the percentage of the weight gain and specific growth rate of red tilapia (*Oreochromis mossambicus* × *Oreochromis niloticus*), reduced the feed conversion ratio, increased hepatic and intestinal protease and amylase activities, and improved digestive performance [42].

The hepatopancreas is an important digestive organ in crayfish, with functions such as detoxification and the prevention of liver damage [43]. AST and ALT are amino–aminotransferases mainly found in liver cells, which are important for the metabolic activities of fish and shrimp, and the levels of AST and ALT become high only when there is damage to the liver [44,45]. In this experiment, it was found that the 1FMO group of the crayfish had the lowest activities of AST and ALT, proving that the addition of 1% fermented *M. oleifera* leaves to the crayfishes’ diets could better reduce liver damage. Crayfish have a poor immune system and usually rely only on non-specific immunity to fight pathogens, and the activities of SOD, CAT, GSH-Px, and MDA enzymes are important indicators to evaluate non-specific immunity in crayfish [46]. Jerimoth et al. (2023) demonstrated that adding 1% Moringa leaves to feed can enhance the serum immunity and improve the gut microbiota structure of catfish [19]. In this experiment, by measuring several classical non-specific immune indicators, the highest antioxidant enzymatic activity was found in the 1FMO group, indicating that the addition of 1% fermented *M. oleifera* leaves has a certain effect on improving the antioxidant capacity of crayfish. Some related studies have shown that the fermentation of feed can significantly increase the nutrient content and produce a variety of antioxidant substances, which are important for improving the growth performance and antioxidant capacity and enhancing the immune ability of farmed animals [47]. Similarly, the use of fermented *M. oleifera* leaves to replace 40–60% of the fishmeal in the diet significantly increased the serum’s immune enzyme (SOD, CAT, and lysozyme (LZM)) activities and C3 levels and reduced serum MDA levels in *Carassius auratus* [5].

Protecting the intestinal health of aquatic animals is an important function of fermented feeds. On one hand, after animals ingest fermented feeds, beneficial bacteria colonize the intestinal tract, compete with pathogenic bacteria for ecological sites, and secrete antimicrobial peptides, bacteriocins, etc. to inhibit the proliferation of pathogenic bacteria, thus achieving the goal of improving the structure of the animal’s intestinal microbiome [48]. On the other hand, metabolites secreted by probiotics after colonization of the gut, such as short-chain fatty acids (SCFAs) and lactic acid, have been shown to repair the host’s intestinal mucosa, build an intestinal barrier, and improve the host’s resistance to pathogenic bacteria [49,50]. In this experiment, the results of the LEfse analysis of variance showed that *Aeromonas hydrophila* was the main variant in the control group and that *A. hydrophila* is a major infectious agent that can cause septicemia in aquatic animals [51]. Compared with the control and 2FMO groups, the 1FMO group significantly decreased the abundance of *Aeromonas* and increased the ratio of (Firmicutes + Bacteroidetes)/Proteobacteria, and the PCA results showed that the 1FMO group improved the structure of the intestinal microbiome. The above results suggest that the addition of 1% fermented *M. oleifera* leaves has a promotive effect on the intestinal health of crayfish. Consistent with our results, Zhang et al. (2021) showed that fermented feeds increased the abundances of intestinal Firmicutes, *Mycobacterium*, *Bacillus*, and *Lactobacillus* and reduced the abundance of *Vibrio* in the intestinal tract of *Penaeus vannamei* [47]. The KEGG enrichment analysis showed that the three experimental groups were mainly focused on metabolic pathways, and the secondary pathway results showed that the addition of 1% fermented *M. oleifera* leaves to the diets increased the expression levels of signaling molecules and interactions—transcriptional, infectious disease: parasitic and infectious disease: viral pathways—compared to those of the control group. Antioxidant enzymes, such as SOD, scavenge ROSs in the cytoplasm and the SOD activity is an indicator of the body’s non-specific immune function. MDA is one of the end products of free-radical lipid peroxidation damage and is cytotoxic, reflecting the extent of peroxidative damage to the body [52]. Correlational analysis at genus level showed that T-AOC, SOD, GSH-Px, and CAT were negatively correlated with *Aeromonas*, but MDA was significantly positively correlated with *Aeromonas*. It was demonstrated that the accumulation of *Aeromonas* could lead to free-radical lipid peroxidation and reduce the antioxidant capacity of crayfish, resulting in oxidative damage to the crayfish organism and lowering its immunity.

## 5. Conclusions

**Figure 12:**
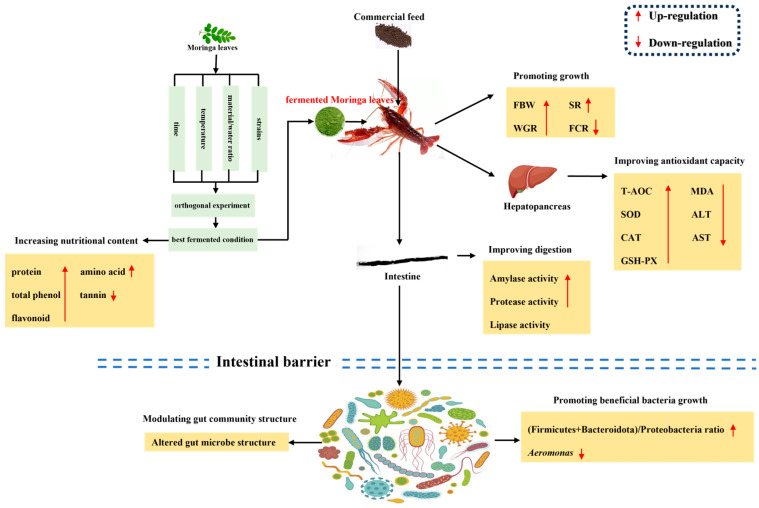
The possible health-promoting effects of fermented *M. oleifera* leaves on the crayfish in vivo.

In conclusion, the optimal fermentation conditions for *M. oleifera* leaves were the fermentation strain of *B. amyloliquefaciens* JY-24 (10^7^ CFU mL^−1^), a material/water ratio of 10%, a fermentation time of 48 h, and a fermentation temperature of 30 °C. These resulted in the production of a high-protein, high-total-phenol, high-amino-acid, and low-tannin product for *M. oleifera* leaves. According to the above experimental results, we summarize the positive effects of fermented *M. oleifera* leaves on crayfish (Figure 12). Briefly, the incorporation of 1% fermented *M. oleifera* leaves improves the growth of the crayfish by increasing the antioxidant capacity of the hepatopancreas, increasing the digestive capacity of the intestine, inhibiting the abundance of pathogenic bacteria, and promoting the growth of value-added beneficial intestinal bacteria. Nevertheless, this experiment was deficient in mechanistic studies on crayfish, and further research is required to explore the functional mechanism of the antioxidant capacity of fermented *M. oleifera* leaves.

## Figures and Tables

**Table 1:** Orthogonal test of fermented *M. oleifera* leaves.

Number	Factors			
	Strain A	Material/Water Ratio (%) B	Temperature (°C) C	Time (h) D
1	1 (S1X-15)	1 (7.5%)	1 (30 °C)	1 (24 h)
2	1 (S1X-15)	2 (10.0%)	2 (35 °C)	2 (48 h)
3	1 (S1X-15)	3 (12.5%)	3 (40 °C)	3 (72 h)
4	2 (IIIA-2)	1 (7.5%)	2 (35 °C)	3 (72 h)
5	2 (IIIA-2)	2 (10.0%)	3 (40 °C)	1 (24 h)
6	2 (IIIA-2)	3 (12.5%)	1 (30 °C)	2 (48 h)
7	3 (JY-24)	1 (7.5%)	3 (40 °C)	2 (48 h)
8	3 (JY-24)	2 (10.0%)	1 (30 °C)	3 (72 h)
9	3 (JY-24)	3 (12.5%)	2 (35 °C)	1 (24 h)

**Table 2:** Ingredients and approximate compositions of the basal diets.

Ingredient	Diet				
	CT (Basal Diet)	0.25FMO (Basal Diet + 0.25FMO)	0.5FMO (Basal Diet + 0.5FMO)	0.5FMO (Basal Diet + 1FMO)	2FMO (Basal Diet + 2FMO)
Domestic fish meal	5.0	5.0	5.0	5.0	5.0
Soybean meal	25.0	25.0	25.0	25.0	25.0
Rapeseed meal	18.0	18.0	18.0	18.0	18.0
Shrimp meal	3.0	3.0	3.0	3.0	3.0
Peanut meal	5.0	5.0	5.0	5.0	5.0
Spray-dried blood cell powder	5.0	5.0	5.0	5.0	5.0
Domestic DDGS	3.0	3.0	3.0	3.0	3.0
α-starch	18.79	18.54	18.29	17.79	16.79
Rice bran	4.0	4.0	4.0	4.0	4.0
Soybean oil	3.0	3.0	3.0	3.0	3.0
MCP	2.0	2.0	2.0	2.0	2.0
Premix	2.0	2.0	2.0	2.0	2.0
Carboxymethyl cellulose	0.5	0.5	0.5	0.5	0.5
Salt	0.3	0.3	0.3	0.3	0.3
Ecdysone	0.01	0.01	0.01	0.01	0.01
Squid paste	3.0	3.0	3.0	3.0	3.0
Choline chloride	0.5	0.5	0.5	0.5	0.5
Calcium bicarbonate	1.5	1.5	1.5	1.5	1.5
Methionine	0.4	0.4	0.4	0.4	0.4
*M. oleifera*	0	0.25	0.5	1	2
Total	100	100	100	100	100
Nutrient composition					
Crude protein	29.94%	29.94%	29.94%	29.94%	29.94%
Crude lipid	9.05%	9.05%	9.05%	9.05%	9.05%
Moisture	12.43%	12.43%	12.43%	12.43%	12.43%

Notes: (1) Fish oil and (2) soybean oil were obtained from Wuxi Tongwei Feedstuffs Co., Ltd. (Wuxi, China). (3) Vitamin and mineral premix (IU, g, or mg kg^−1^ of the diet): vitamin A, 25,000 IU; vitamin D3, 20,000 IU; vitamin E, 200 mg; vitamin K3, 20 mg; thiamin, 40 mg; riboflavin, 50 mg; calcium pantothenate, 100 mg; pyridoxine HCl, 40 mg; cyanocobalamin, 0.2 mg; biotin, 6 mg; folic acid, 20 mg; niacin, 200 mg; inositol, 1000 mg; vitamin C, 2000 mg; choline, 2000 mg; calcium biphosphate, 20 g; sodium chloride, 2.6 g; potassium chloride, 5 g; magnesium sulfate, 2 g; ferrous sulfate, 0.9 g; zinc sulfate, 0.06 g; cupric sulfate, 0.02 g; manganese sulfate, 0.03 g; sodium selenate, 0.02 g; cobalt chloride, 0.05 g; potassium iodide, 0.004 g.

**Table 3:** Extreme variance analysis of fermented *M. oleifera* leaves.

	Strain A	Material/Water Ratio (%) B	Temperature (°C) C	Time (h) D	Protein Content (mg g^−1^)
1	1 (S1X-15)	1 (7.5%)	1 (30 °C)	1 (24 h)	257.6 ± 28.12
2	1 (S1X-15)	2 (10%)	2 (35 °C)	2 (48 h)	211.46 ± 18.59
3	1 (S1X-15)	3 (12.5%)	3 (40 °C)	3 (72 h)	128.63 ± 18.59
4	2 (IIIA-2)	1 (7.5%)	2 (35 °C)	3 (72 h)	77.04 ± 8.72
5	2 (IIIA-2)	2 (10%)	3 (40 °C)	1 (24 h)	192.32 ± 34.18
6	2 (IIIA-2)	3 (12.5%)	1 (30 °C)	2 (48 h)	205.16 ± 4.64
7	3 (JY-24)	1 (7.5%)	3 (40 °C)	2 (48 h)	213.84 ± 13.93
8	3 (JY-24)	2 (10%)	1 (30 °C)	3 (72 h)	325.48 ± 29.98
9	3 (JY-24)	3 (12.5%)	2 (35 °C)	1 (24 h)	171.43 ± 18.05
k_1_	579.69	548.48	788.24	621.35	
k_2_	474.96	729.26	459.93	630.46	
k_3_	710.75	505.22	534.79	531.15	
R	235.79	224.04	328.31	99.31	
Primary–secondary factors	C > A > B > D				
Optimal combination	C_1_ A_3_ B_2_ D_2_				

Note: A: strains; B: ratio of material/water; C: temperature; D: time; k_1–3_: the influence of each factor on the test index; R: the influence of each factor on the test index is reflected.

**Table 4:** Changes in nutrient composition and tannin content of fermented *M. oleifera* leaves.

	Before Fermentation	After Fermentation	*p*-Value
Total protein (mg g^−1^)	314.71 ± 28.41	416.53 ± 20.75	0.009
Total phenols (mg g^−1^)	6.93 ± 2.49	39.57 ± 4.12	0.001
Flavonoids (mg g^−1^)	0.32 ± 0.03	0.78 ± 0.09	0.007
Amino acids (µmol g^−1^)	235.58 ± 14.58	369.84 ± 4.23	0.002
Tannins (nmol mg protein^−1^)	0.22 ± 0.02	0.20 ± 0.005	0.012

**Table 5:** Effect of fermented *M. oleifera* leaves on growth performance of crayfish.

	CT	0.25FMO	0.5FMO	1FMO	2FMO
IBW (g)	9.14 ± 0.03 ^a^	9.07 ± 0.06 ^a^	9.12 ± 0.08 ^a^	9.13 ± 0.12 ^a^	9.10 ± 0.03 ^a^
FBW (g)	21.43 ± 1.85 ^bc^	19.62 ± 1.33 ^c^	23.10 ± 1.51 ^ab^	25.62 ± 1.67 ^a^	23.02 ± 1.32 ^ab^
FCR	1.73 ± 0.03 ^a^	1.55 ± 0.11 ^a^	1.53 ± 0.46 ^a^	1.63 ± 0.25 ^a^	1.97 ± 0.81 ^a^
WGR (%)	134.43 ± 19.48 ^bc^	116.15 ± 13.26 ^c^	153.44 ± 18.77 ^b^	180.41 ± 14.65 ^a^	152.94 ± 13.53 ^ab^
SGR (%)	1.70 ± 0.17 ^bc^	1.54 ± 0.13 ^c^	1.86 ± 0.15 ^ab^	2.06 ± 0.10 ^a^	1.86 ± 0.11 ^ab^

Note: Different letters in the different groups indicate significant differences, *p* < 0.05. WGR (%) = (G_2_ − G_1_)/G_1_ × 100; SGR (%/d) = (lnG_2_ − lnG_1_)/D × 100; FCR = G_3_/G’. G_1_ is the average initial weight of the crayfish (g), G_2_ is the average final weight of the crayfish (g), D is the duration of the culture trial (days), G_3_ is the total food intake (g), and G’ is the total weight gain.

## Data Availability

The original contributions presented in this study are included in the article. Further inquiries can be directed to the corresponding author(s).
